# Molecular polymorphism of tau aggregates in Pick’s disease

**DOI:** 10.1016/j.nbd.2025.107104

**Published:** 2025-09-14

**Authors:** Jiliang Liu, Theresa Connors Stewart, Derek H. Oakley, Bradley T. Hyman, Manfred Burghammer, Marine Cotte, Lee Makowski

**Affiliations:** a European Synchrotron Radiation Facility, Grenoble 38043, France; b Massachusetts Alzheimer’s Disease Research Center, Boston, MA, USA; c Department of Pathology, Massachusetts General Hospital, Boston, MA,USA; d C.S. Kubik Laboratory for Neuropathology, Massachusetts General Hospital, Boston, MA, USA; e Department of Neurology, Harvard Medical School, Boston, MA, USA; f Bioengineering Department, Northeastern University, Boston, MA 02115, USA

**Keywords:** Tau, Pick’s disease, Pick bodies, X-ray microdiffraction, X-ray fluorescence microscopy

## Abstract

**Background:**

Tau protein is central to progressive neuropathological changes in many neurodegenerative diseases. Although the trajectory by which tau pathology spreads through neural networks has been studied, the molecular processes that drive disease are unknown. Characterizing these processes requires imaging tools that supplement neuropathological examination with information about the molecular organization of lesions while preserving details of their anatomical location. High-resolution cryo-electron microscope studies require isolation of material which destroys information on variation of lesion structure with location. Immunohistochemistry identifies principal constituents of lesions but provides little information about molecular organization. To bridge this knowledge gap, we have developed advanced biophysical imaging tools to probe, *in situ*, the molecular organization and composition of individual lesions within diseased brain tissue. Here we describe their use to assess the fibrillar organization and variation of elemental composition in tau-containing lesions within the brain of a 66-year-old male with dementia.

**Methods:**

We used *in situ* micro X-ray diffraction (μXRD) to determine the aggregation state of tau in individual lesions and micro-X-ray fluorescence (μXRF) to determine the elemental content of these lesions. The information thus generated was combined with immunohistochemistry of serial sections that confirmed the principal molecular constituents of lesions and placed the results in a broader anatomical context.

**Results:**

Neuropathological examination revealed classical forms of tau inclusions in the hippocampal formation including extensive Pick bodies in the dentate gyrus. μXRD data indicate that Pick bodies of the granular layer are relatively low in fibril content, whereas, surprisingly, the microscopically diffuse tau in the neuropil in adjacent CA4 and hilus regions exhibit far greater density of fibrillar signal. μXRF data show elevated levels of zinc, calcium and phosphorous relative to surrounding tissue in essentially all tau-containing lesions. Sulfur deposition appeared greater in areas exhibiting high fibrillar content. A second case of Pick’s disease showed analogous results.

**Conclusions:**

These observations demonstrate a correlation of lesion morphology with anatomical localization, degree of tau fibrillation and differential accumulation of metals and suggest that lesions containing different levels of tau fibrils harbor biochemically distinct microenvironments.

## 1. Introduction

Pick’s disease, a subtype of frontotemporal lobar degeneration with tau pathology (FTLD-tau), is a progressive, neurodegenerative disorder that impacts the brain’s frontal and temporal lobes. Aggregates composed of tau protein are closely associated with the progression of dementia in Pick’s disease. Pick bodies, intracellular spherical inclusions unique to Pick’s disease, build up inside neurons that take on a roughly spherical shape. Within these lesions, fibrillar forms of tau contain only 3R isoforms and exhibit a unique cross-sectional core structure. (Falcon et al., 2018)

*In situ*, in addition to the well studied cross-β fibrils, tau aggregates may exhibit relatively diverse morphologies including oligomeric forms, amorphous or ring-like aggregates, small oligomers, or monomeric species. (Mate De Gerando et al., 2023) Most studies of the molecular structure of these aggregates utilize purified tau and cannot provide positional information on structural variation within tissue. But the spatial distribution of fibrillar and smaller aggregates of tau in tissue may well influence the processes underlying seeding and spreading of tau. (Mate De Gerando et al., 2023) Therefore, generating information on the positional variation of aggregate structure is a high priority. Here, we use scanning micro X-ray diffraction (μXRD) to study the structural variations of tau within tissue and micro X-ray fluorescence (μXRF) microscopy to assess the accumulation of specific elements within the lesions. In this approach, a histological tissue section is raster scanned in front of a micro-focused X-ray beam and μXRD and μXRF data are collected simultaneously to characterize position-specific variation of molecular structure and biometal accumulation with subcellular resolution. Immunohistochemistry of serial sections is used to identify the major constituents of lesions.

The size and shape of pathological lesions in human brain tissue can be determined by mapping the intensity of small and wide-angle X-ray scattering across a thin section. (Liu et al., 2016) Higher intensity indicates denser packing of macromolecular constituents and most pathological lesions appear to have higher packing density than the surrounding proximal tissue. The size and shape of features identified in this way correspond closely with those of tau-staining lesions observed by immunohistochemistry in the associated serial sections, providing strong evidence of their identity as tau-rich lesions. Other structural features of tissue, such as vascular walls also have greater density than the surrounding tissue and these can be identified by their characteristic shapes in maps of scattering intensity.

All neuropathological fibrils have a distinctive cross-β fibrillar structure which gives rise to a stereotypic X-ray scattering ‘fingerprint’. In a cross-β structure, β-strands of protein extend across the fibril, perpendicular to the fibril axis, forming β-sheets of indeterminant length. The structure acts as a diffraction grating, generating strong X-ray scattering at angles that correspond to the axial distance between β-strands (4.7 Å) and the transverse distance between the β-sheets (10 Å). (Berriman et al., 2003; Kirschner et al., 1986) X-ray scattering from a tissue sample measured with conventional diffractometers will not produce high quality signal from fibrils embedded in the tissue because the scattering volume is so large as to encompass heterogeneous tissue structures beyond the boundaries of the lesion. This results in scattering patterns that are the sum of scattering from a heterogeneous mixture of structures that obscures the scattering from lesions and precludes interpretation on the basis of individual molecular structures. The use of a microbeam from a synchrotron source allows collection of a scattering signal from a volume sufficiently small that it falls entirely within a lesion and is dominated by a single constituent, thereby giving rise to scattering patterns that can be interpreted in terms of the structure of that constituent. (Carboni et al., 2017; Joppe et al., 2025; Araki et al., 2015) This makes possible study of the molecular structure of tau aggregates such as Pick bodies that vary in size from a few microns to tens of microns. Correlation with immunohistochemistry of serial sections makes it possible to positively identify the principal constituents of these lesions and to place the structural attributes of these aggregates in the broader context of the cellular organization of the tissue.

As will be demonstrated here, data in the wide-angle portion of the X-ray patterns (WAXS) can be used to assess the degree of fibrillation of tau, while intensity in the small angle X-ray scattering regime (SAXS) can provide insights into the structure and packing of tau fibrils. Simultaneous collection of X-ray fluorescence (XRF) data makes possible correlation of the deposition of selected metal and non-metal elements with the different levels of tau accumulation and fibrillation revealed by the scattering data. When combined with correlated immunohistochemistry of serial sections, these data provide a basis for associating distinct tau structures with different cell types distributed within specific regions of the brain. The structural information derived directly from tissue can provide insights in structure of tau in Pick’s disease to supplement that derived through studies of isolated materials.

## 2. Methods

### 2.1. Sample preparation

Brain tissue was prepared at the Massachusetts Alzheimer’s Disease Research Center (MADRC). Tissue was formalin fixed using standard neuropathological processes and sectioned to a thickness of 20 μm as previously described. (Liu et al., 2016) The brains collected by MADRC are handled, dissected, and stored in a uniform fashion. Brains are divided in half with one half fixed, processed in paraffin for neuropathological analysis and immunohistochemical studies and the other half generally snap-frozen in coronal slices. Regions of interest are cut from the fixed hemisphere, blocked into cassettes and stored in 10 % formalin. Once selected for examination, a block is soaked in 98 % formic acid for one hour. A tissue processor is then used to wash the tissue with formalin, and then a series of ethanol and then xylene solutions overnight. The tissue is then embedded in paraffin and cooled to harden the wax. Tissue is held at 5 °C for sectioning into 20 μm thick sections. Three serial sections are collected, including one that is left unstained for x-ray analysis, one for immunostaining for tau and one for Aβ.

Unstained sections were prepared thicker than conventional histological sections in order to increase the volume of material irradiated thereby improving the signal-to-noise ratio of the scattering patterns collected. Tissue sections were mounted on 5 × 5 mm^2^ 1 μm thick Si_3_N_4_ membranes and affixed to sample holders customized for the ID13 beam line at ESRF.

### 2.2. Tissue selection

Analysis was carried out on tissue from two subjects. Subject 1 was a 66-year-old man initially presented at age 60 with increasingly progressive behavioral changes consistent with behavioral variant of frontotemporal dementia (FTD). Neuropathological evaluation demonstrated the underlying etiology to be Pick’s disease (FTLD-tau). Sections showed severe cortical degeneration as well as numerous tau positive Pick bodies and swollen tau positive neurons (Pick cells), especially in the dentate gyrus and present also in the frontal and temporal lobes. These changes are consistent with the Pick’s disease sub-category of frontotemporal dementia (FTLD-tau). Subtle, diffuse tau immunostaining of the neuropil as well as numerous neuritic threads, were present in all hippocampal subfields including the hilus. There were substantial neuropil tau containing threads, and incidental diffuse sparse, non-neuritic β-amyloid plaques primarily in cortical areas.

Subject 2 was a 79-year-old man had a clinical history consistent with frontotemporal lobar degeneration (FTLD). Neuropathologic evaluation revealed the underlying etiology to be Pick’s disease (FTLD-Tau). Sections showed severe cortical degeneration as well as numerous tau-positive (AT8) Pick bodies and swollen tau-positive neurons (Pick cells) predominantly in the frontal and temporal lobes with less prominent extension of tau pathology into the parietal and occipital lobes. Prominent neuropil diffuse tau was observed in the hippocampal formation. Concurrent sparse β-amyloid neuritic plaques and neurofibrillary tangles were also observed in the entorhinal cortex and other neocortical areas.

Results on the analysis of Subject 1 are in the main text; results on Subject 2 are included in the Supplementary Material.

### 2.3. Simultaneous collection of μXRD and μXRF maps

**Fig. 1.**
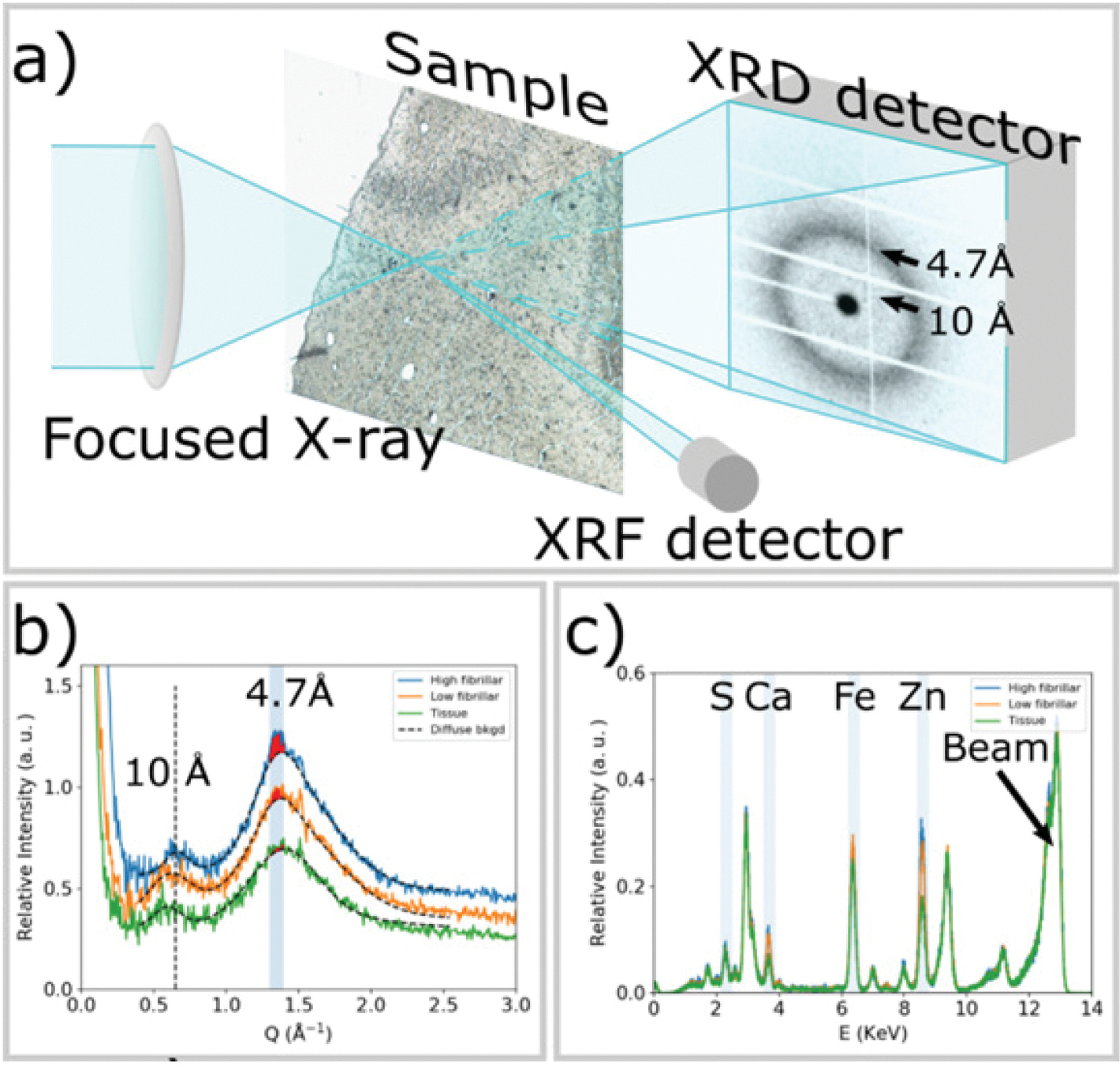
**a)** Experimental scheme for collecting μXRD and μXRF data simultaneously. The X-ray beam is focused to 2.5 μm by the lens and the sample is raster scanned with step size of 2.5 μm. μXRD from tau lesions gives rise to a prototypical cross-β fiber diffraction pattern, which is dominated by reflections at scattering angles corresponding to periodicities of 10 Å and 4.7 Å. An XRF detector is placed nearly perpendicular to the sample to simultaneously collect XRF signal. **b)** Azimuthally averaged scattering patterns from a tau lesion with significant high-fibrillar tau (blue); a tau-containing lesion containing low-fibrillar tau (orange) and a region of tissue exhibiting no tau pathology (green). High-fibrillar tau gives rise to a pronounced 4.7 Å peak, the intensity of which can be estimated by subtracting a smooth background and integrating the remaining intensity (red fill). Short fibrils, or tau aggregates that are low-fibrillar may give rise to a weak peak at 4.7 Å spacing that can also be estimated through subtraction of a smooth background. Surrounding tissue also gives rise to broad scattering peaks at ~10 Å and 4.7 Å spacing, but lacks the additional pronounced features at 4.7 Å. **c)** μXRF spectra from a tau lesion with significant high-fibrillar tau (blue); a tau-containing lesion with little or low-fibrillar tau (orange) and a region of tissue exhibiting no tau pathology (green). The spectral line exhibits patterns of chemical elements, including sulfur (S) with K_α_ edge at 2.31 KeV, calcium (Ca) at 3.64 KeV, iron (Fe) at 6.4 KeV and zinc (Zn) at 8.64 KeV. The peak at 13 KeV arises from Rayleigh and Compton scattering of the incident X-ray beam. The maps of elemental distributions are calculated by integrating the spectrum over a range of ± 0.1KeV around the corresponding K_α_ energy for each pixel. Intensity of peaks in the XRF spectrum reveal a relative degree of deposition of each element within the tissue sample.

A summary of the data collection process is in Fig. 1. Scanning μXRD was conducted at beam line ID13, at the European Synchrotron Radiation Facility (ESRF). The monochromatic beam was focused to a Gaussian profile with FWHM of 2.5 μm in both the horizontal and vertical directions, using compound refractive lenses. To reduce secondary radiation damage, a fly scanning mode was applied with scan step of 2.5 μm across a square grid. The X-ray beam is monochromatic, having an energy of 13 KeV and a flux of 2.5 × 10^12^ photons/s. The exposure time was 50 msec. The microdiffraction patterns were collected in transmission mode by an Eiger 4 M detector.

XRF spectra from elements heavier than calcium including zinc were collected with a Vortex EM detector installed almost normal to the path of the X-ray beam and pointing in the direction of the sample (Fig. 1). Data processing was conducted using a custom program developed for scanning μXRD. XRF spectra have been analyzed using the PyMca program. (Solé et al., 2007)

### 2.4. Mapping distribution of tau aggregates

The distribution of tau lesions within a fixed thin section was mapped using the intensity of scattering integrated over the range (0.05 Å^−1^ < Q < 0.25 Å^−1^). Intensity in this regime is approximately proportional to the electron density contrast of the macromolecular material within the scattering volume. When scanning a section of uniform thickness this is associated with the density of material in the tissue. There is substantial evidence that the density of macromolecular scattering material is greater in lesions (Aβ or tau) than in the surrounding material. (Liu et al., 2016; Bashit et al., 2022) Other anatomical features such as vascular walls also have greater density than the surrounding tissue, and these are usually readily identified from their size and shape. Consequently, a map of scattering intensity across an ROI will allow the distribution of pathological lesions to be derived. The intensity in the range (0.05 Å^−1^ < Q < 0.25 Å^−1^) was used to calculate the maps of molecular density shown in the Results section because the intensities and signal-to-noise ratio in that Q-range are greater than at larger Q. Scattering intensities at smaller angles are impacted by scattering from voids formed during tissue processing and dehydration (Nepal et al., 2024) and cannot be used for this purpose.

### 2.5. Mapping the distribution of tau fibrils

Cross-β structure gives rise to a pronounced peak at ~4.7 Å spacing (Q = 1.34 Å^−1^). As diagrammed in Fig. 1b, the relative abundance of fibrillar tau was estimated by subtracting the broad diffuse background scattering and integrating the difference intensity over the Q range −1.32 Å^−1^ to 1.4 Å^−1^. The smooth background curves (dashed lines) are calculated as a sum of Pseudo-Voigt functions and represent an estimate of the scattering of tissue, which is comprised largely of fixed, partially denatured macromolecules. By subtracting the simulated broad tissue background (dashed curves in Fig. 1b) from the data, the degree of tau fibrillation can be estimated from the magnitude of the residual intensity, highlighted in red in Fig. 1b. Thus, scanning μXRD data enable us to map both the distribution of tau and the degree of tau fibrillation *in situ* across histological sections with micron-level resolution, making possible the correlation of structural information at the molecular level with cellular and tissue distributions.

### 2.6. High resolution X-ray fluorescence imaging of light elements

Lighter elements such as S and P could not be accurately estimated at ID13 where XRF is excited at 13 keV (far from their absorption edges) and XRF is collected in air (high reabsorption of low energy XRF signal). To determine the distribution of lighter elements, the same sample was reanalyzed in vacuum at beam line ID21 at ESRF. (Cotte et al., 2017) For these experiments, the beam was focused to 0.3 μm (vertical) × 0.8 μm (horizontal) using Kirckpatrick Baez mirrors. Maps were acquired over regions of interest (ROI) of 99 μm × 99 μm, with steps of 1 μm, and exposure time of 200 ms. The X-ray beam was tuned to an energy of 4KeV to optimize signal from light elements including S, P and *Ca.* The use of a different sample holder for the ID13 and ID21 experiments precluded exact registration of the ID21 μXRF scans with the ID13 μXRD scans of the same sections. However, mapping of Ca distribution was carried out on both beam lines. This made it possible to use the Ca distribution collected at low X-ray energy as a guide to registration of scans taken at high X-ray energy simultaneously with the μXRD data.

## 3. Results

### 3.1. Multimode X-ray imaging

Fig. 1a diagrams the experimental arrangement of multimode X-ray imaging, simultaneously collecting X-ray scattering and X-ray fluorescence data across a tissue section raster scanned in front of a micro-focused X-ray beam. Details about the experimental set-up, sample preparation and data processing are given in the methods section. In brief, thin sections of human brain tissue from a Pick’s disease subject were prepared as previously described (Liu et al., 2016) and spread on 1 μm thick Si_3_N_4_ membranes. These sections were scanned at the micro-end-station of the beamline ID13, at the European Synchrotron Radiation Facility (ESRF) with an X-ray beam having full width half maximum (fwhm) of 2.5 μm. 2D regions of interest (ROI) 1500 μm on a side were defined for each section and XRD and XRF data were collected on a square, 2.5 μm grid across these ROIs (600 × 600 frames constituting 360,000 diffraction patterns). X-ray patterns from tissue sections are usually circularly symmetric, or nearly so. Consequently, we circularly averaged the patterns to improve signal-to-noise ratio. As shown in Fig. 1b, the circularly averaged data includes strong intensity in the small-angle (SAXS) regime and distinctive broad peaks at scattering angles that correspond to periodicities of 4.7 Å and 10 Å. Fixed tissue from many organs will exhibit broad peaks at these spacings due to residual secondary structure in partially denatured proteins. Scattering from regions rich in fibrillar material (blue curve in Fig. 1b) gives rise to a relatively sharp, pronounced reflection at 4.7 Å spacing located on a broad scattering peak arising from tissue constituents interspersed with the fibrils. Lesions with low fibrillar content produce a relatively weak 4.7 Å peak (orange curve). Tissue regions devoid of tau fibrils or aggregates generate only a broad wide-angle peak (green curve) that is usually weaker than that observed from tau-containing lesions. Analysis of the scattering patterns in terms of the structure of the molecular constituents of the scattering volume is described in Fig. S1. Additional experimental and computational details are given in the methods section. Simultaneously collected XRF signal identified increased fluorescence intensities of calcium, iron and zinc associated with lesions containing tau as seen in Fig. 1c.

### 3.2. Immunostaining of Aβ and tau in Dentate Gyrus

Although tau is present throughout the cortex in Pick’s disease, the dentate gyrus (DG) of the hippocampus frequently contains a high density of tau lesions. (Kawles et al., 2021) The DG granule cell layer appears relatively resilient to the classic hallmarks of Alzheimer’s disease (plaques and tangles) in early stage disease, but in Pick’s disease it may contain a number of different types of tau deposits. These include Pick bodies, exhibiting a prototypic round morphology, often heavily deposited in the granular layer; and thin filaments of tau within dystrophic neurites in the hilus.

**Fig. 2.**
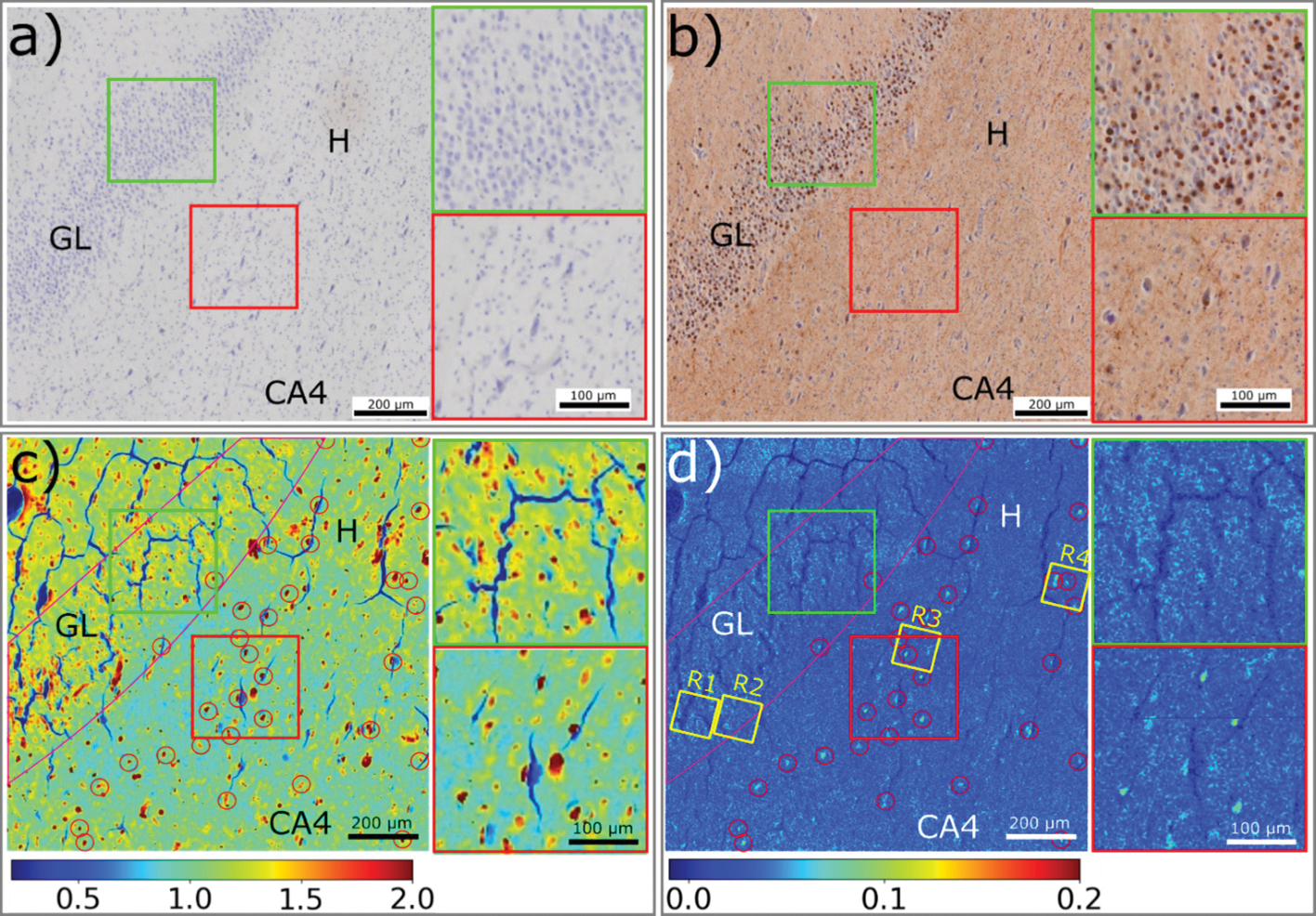
μXRD analysis of a region of a Dentate Gyrus including the Granular Layer (GL), Hilus (H) and the Cornu Ammonis region 4 (CA4) of the hippocampus. **a)** Image of a serial section stained for Aβ corresponding to the region of the unstained section scanned by XRD. Different cell morphologies, including granule and pyramidal cells, are present. **b)** Image of a serial section immunostained for tau (AT8). The granular layer is dominated by Pick bodies, while tau lesions from dystrophic neurites and pyramidal cells are distributed across the hilus region. Two inserts show the distribution of tau lesions in granular layer (green box) and hilus region (red box). **c)** The distribution of macromolecular density is calculated by integrating the small-angle X-ray scattering intensity within the range of 0.05–0.25 Å^−1^ of Q. The distribution of macromolecular density exhibits dense features in both the granular layer, CA4 and hilus and includes features that are low in fibrillar content and those that exhibit a high concentration of fibrillar tau (indicated by red circles). **d)** The distribution of fibrillar tau is determined by the integral intensity of patterns after diffuse background subtraction. Regions containing oligomeric or low fibrillar aggregates appear as light blue; high fibrillar lesions as bright blue (marked by red circles). Two inserts on the right show the distribution of aggregates in the Granular layer (green box) and fibrils in the Hilus region (red box). **R1**-**R4** are four ROIs in which high resolution XRF images were collected as detailed in Fig. 5. Since the scattering intensity correlates to both features of sample and features of X-ray, such as flux and energy, the relative intensity of colour scale for c) and d) is given in arbitrary units. However, the relative intensity is proportional to mass fraction of macromolecular materials (panel c) and fibrillar tau (panel d) within the beam path.

Fig. 2 compares images of the tissue produced by different imaging modalities. Fig. 2a is an optical micrograph of a portion of the dentate gyrus immunostained for Aβ, but showing little evidence for the presence of Aβ lesions. Fig. 2b is the corresponding serial section immunostained for tau that reveals an abundance of Pick bodies in the granular layer. These are identified by their dark brown staining and round shape as highlighted in the upper inset. The hilus Cornu Ammonis region 4 (CA4) is composed largely of pyramidal cells. (Amaral, 1978; Woelfle and Boeckers, 2021) Fig. S2a provides a wider field of view of the serial section immunostained for tau showing two segments of the granular layer separated by the hilus region that connects to the CA4 and CA3 regions of the hippocampus. The granular layer exhibits particularly dense deposition of Pick bodies. In about 10 % of Pick’s disease cases, including those we report on here, there are features of concurrent Alzheimer’s disease pathology which may include fibrillar tau found inside neuronal cells (including those in the hilus, adjacent to the granule cells of the DG). (Hyman et al., 1988)

### 3.3. The aggregation state of tau is specific to cellular environment

The location of tau lesions, be they Pick bodies, neurofibrillary tangles or dystrophic neurites can be distinguished by the intensity distribution of X-ray scattering in the small/wide-angle regime. As shown in Fig. 1b, the wide-angle scattering can be divided into two classes – broad, diffuse scattering which all proteinaceous macromolecular structures contribute to; and pronounced 4.7 Å scattering which is characteristic of cross-β fibrillar structures such as those formed by tau. The broad wide-angle scattering is observed in all fixed tissue but is usually observed to be more intense in Aβ or tau lesions. (Liu et al., 2016; Bashit et al., 2022) This indicates that pathological lesions are more densely packed with macromolecules than the surrounding tissue. Fig. 2c shows the distribution of macromolecular density across the ROI as estimated from the intensity of small angle scattering in the range 0.05 < Q < 0.25 Å^−1^. The size, shape and density of the features apparent in this mapping correlate well with the corresponding features of Pick bodies observed by immunohistochemistry of serial sections as seen in Fig. 2b.

The location of cross-β fibrillar structure can be determined by estimating the intensity of the pronounced 4.7 Å peak (as detailed in Fig. S1) resulting in a map as shown in Fig. 2d. By comparing the dense features in Fig. 2c with the positions of fibrillar lesions (indicated by red circles) in Fig. 2d, the positions of tau lesions with greater or lesser fibrillar content can be determined. Surprisingly, classical Pick bodies do not show a frank β-pleated sheet signal. By contrast, the hilus, which does not contain Pick bodies in this sample shows a high level of β-pleated sheet structures. While the Pick bodies present in the dentate are a highly specific finding for Pick disease, the tau staining within CA4 may represent Pick disease or concurrent age-related tauopathy, including AD (Dickson et al., 2011; Price et al., 2020). In the CA4 and hilus, fibrillar tau is associated with many but not all lesions. In the granular layer, most lesions exhibit little or no fibrillar structure.

### 3.4. High fibrillar tau exhibits a hierarchical organization

**Fig. 3.**
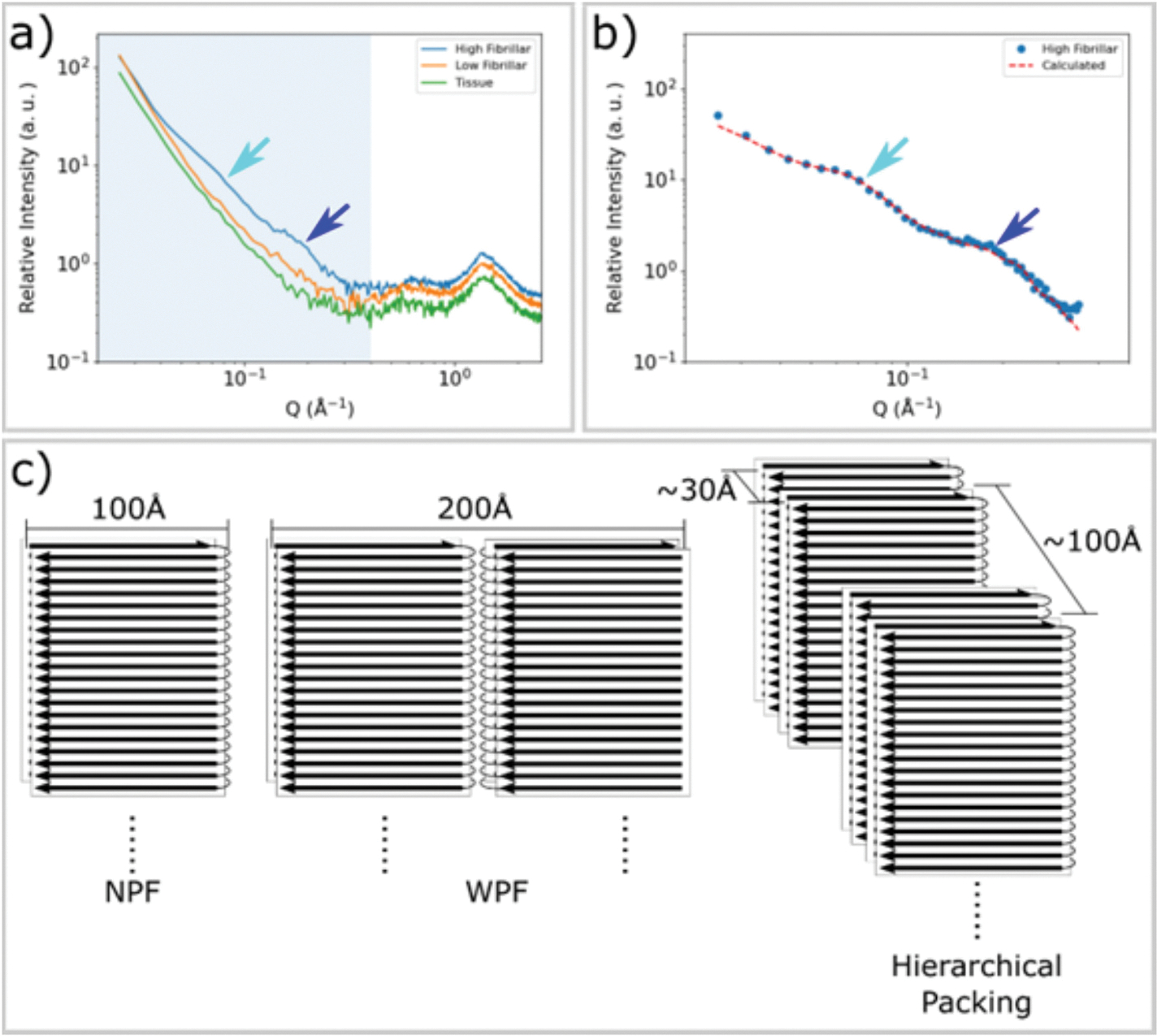
SAXS from fibrillar tau exhibits distinctive peaks. **a)** The SAXS profiles from tissue devoid of tau aggregates (green), containing low fibril content (orange) and having high levels of fibrillar tau (blue) as identified by the intensity of the pronounced WAXS reflection at 4.7 Å spacing. Patterns exhibiting strong cross-β related features in the WAXS regime (Q ~ 1.36 Å^−1^) also exhibit distinctive features in the SAXS regime, highlighted by cyan and blue arrows at Q ~ 0.07 Å^−1^ and ~ 0.2 Å^−1^. **b)** Background-subtracted SAXS intensities correspond well with those calculated from a hierarchical model of fibrillar organization exhibiting hierarchical packing with limited variation in fibril-fibril distances. **c)** A diagram of the narrow Pick filament (NPF), the wide Pick filament (WPF) and the model of hierarchical packing used to fit the observed data. Inter-fibrillar distances of 30 Å and 100 Å are representative of the polymorphic hierarchical organization indicated by the shape of the SAXS scattering as detailed in Fig. S4.

In addition to the 4.7 Å peak, as shown in Fig. 3a, SAXS intensities from fibrillar lesions exhibit features not seen in scattering from tissue or regions of low fibrillar content. Both low fibrillar and high fibrillar tau give rise to stronger SAXS intensity than does tissue, as shown in the mapping of SAXS intensity in Fig. 2c. However, the linear slope of the log-log scaled SAXS profile of low fibrillar tau indicates that the tau and macromolecular aggregates are highly polydisperse in size. In contrast, scattering from regions with a high concentration of fibrils exhibit distinctive peaks or shoulders in the SAXS regime (Fig. 3a). This modulation of SAXS intensity is only observed in patterns that also exhibit pronounced reflections at 4.7 Å spacing, confirming they are due to the presence of cross-β fibrils. These peaks provide information about the transverse (cross-sectional) morphology of the fibrils and their higher-order spatial organization. Quantitative analysis of these fibril-specific features was carried out after subtraction of background from the amorphous tissue and/or voids that are interspersed with fibrils in the scattering volume. (Nepal et al., 2024; Liu and Makowski, 2022)

Simplified structural models for the tau fibril, Fig. 3c, were constructed on the basis of the structure of tau filament cores as determined by cryoEM studies of material extracted from Pick’s disease subjects. (Falcon et al., 2018) However, models based on the structure of individual fibrils (either narrow Pick filament (NPF) or wide Pick filament (WPF)) failed to predict the observed intensities. A simplified cylinder model (Schweers et al., 1994) also failed to reproduce details of the observed experimental intensity. As detailed in the Supplementary Material (Fig. S3 and S4), in order to successfully reproduce the observed intensities, a polymorphic hierarchical organization of fibrils was postulated with characteristic fibril-fibril distances of 30 Å (+/− 15 %) and 100 Å (+/− 30 %). This model resulted in predictions of scattered intensity with characteristics that closely correspond to those observed (Fig. 3b). The relatively large fibril-fibril distances characteristic of this packing appears to be required in order to accommodate the intrinsically disordered ‘fuzzy coat’ of tau (*e.g.* (Fitzpatrick et al., 2017)) that accounts for about 75 % of the mass of the tau molecule in neurofibrillary tangles.

### 3.5. Association of elemental accumulation with aggregation state of tau

**Fig. 4.**
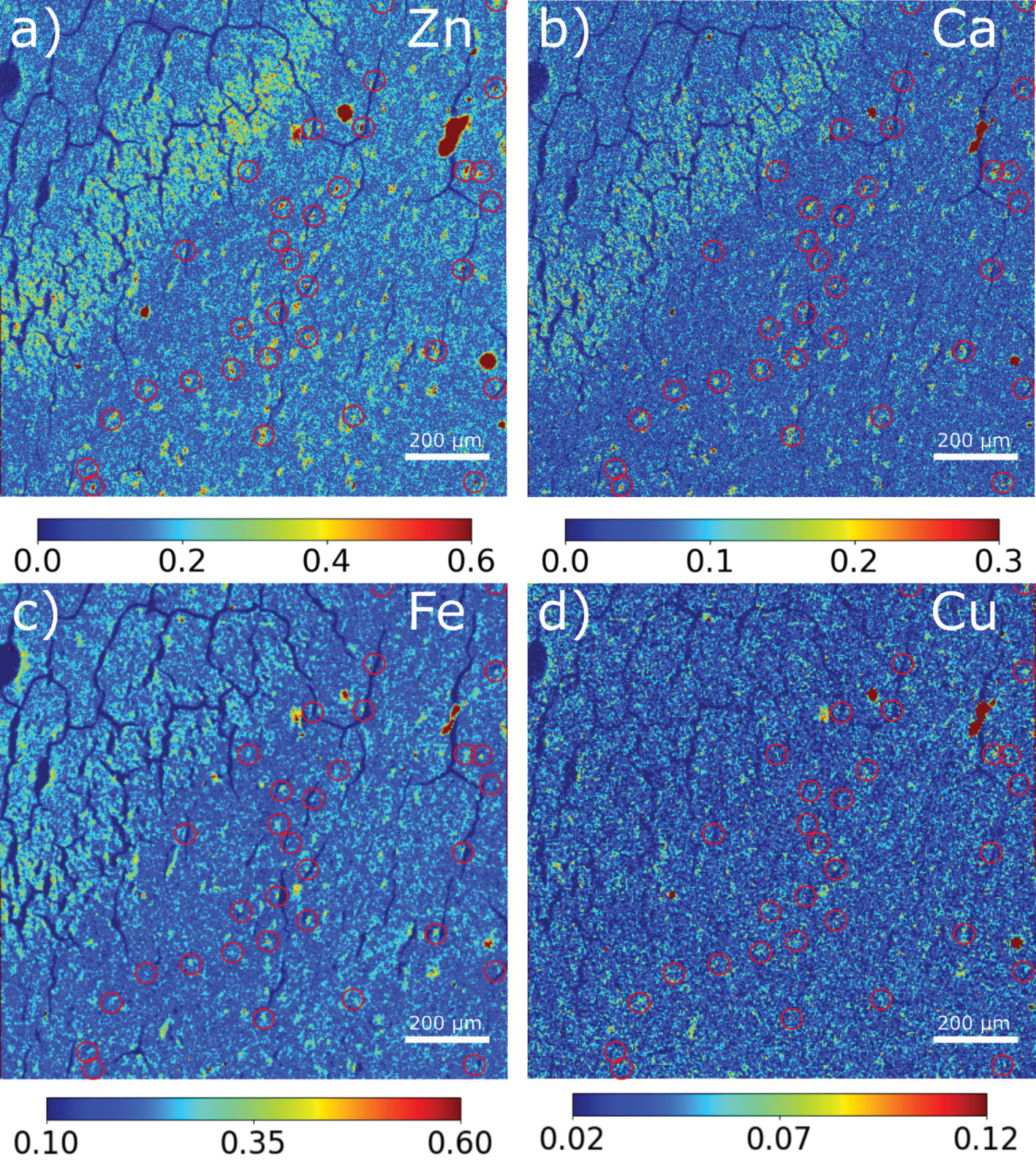
Distribution of X-ray fluorescence signal from **(a)** zinc, **(b)** calcium, **(c)** iron and **(d)** copper. Red circles indicate the locations of fibrils as determined from μXRD. The relative intensity of colour scale bars is in arbitrary units. The relative intensity is proportion to the mass fraction of elemental deposition.

**Fig. 5.**
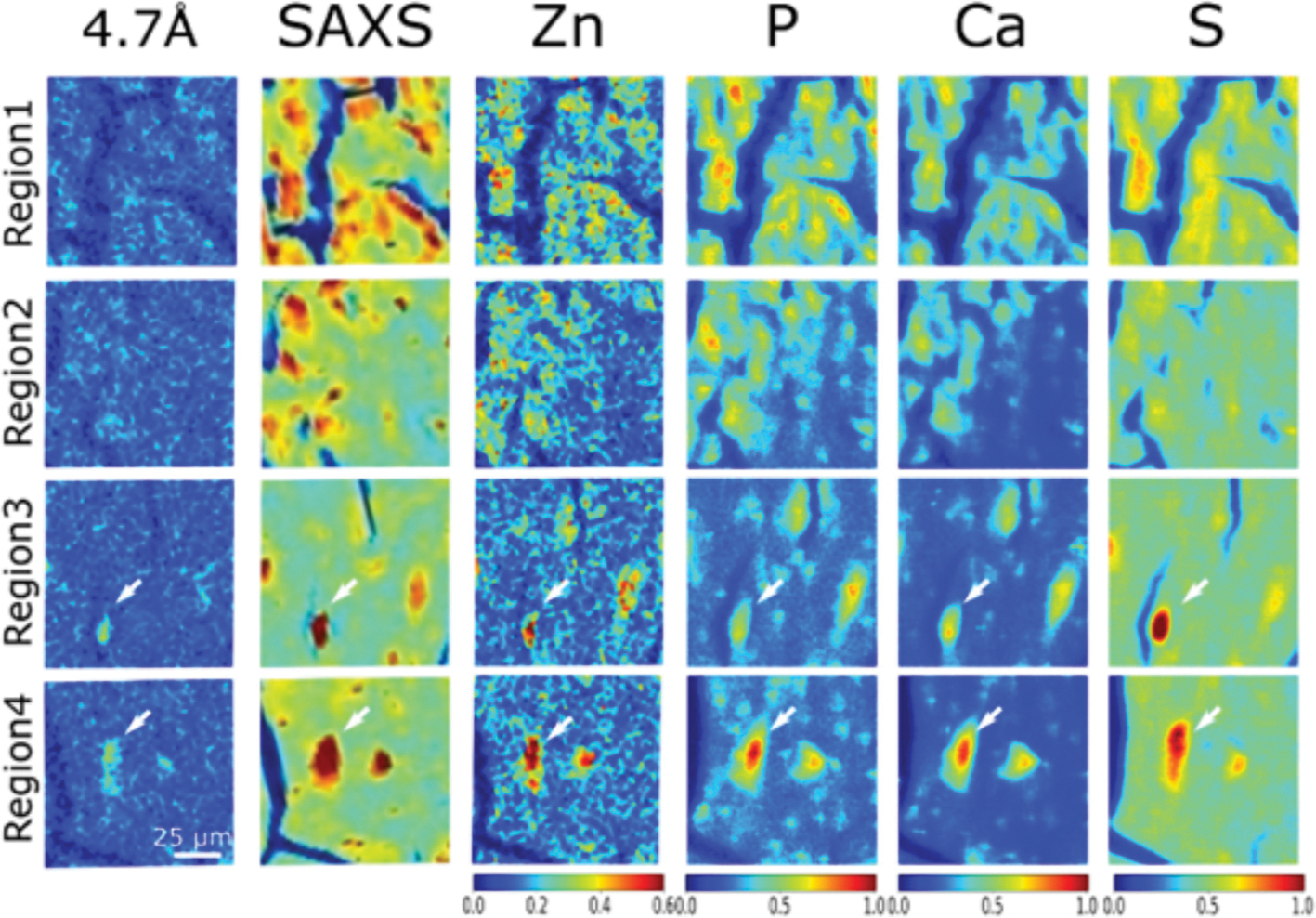
Correlation of the distribution of tau fibrils (left column), integral intensity of SAXS from macromolecular deposition (second column from left) and distribution of select elements. **Regions 1–4** correspond to those highlighted as yellow boxes in Fig. 2d and **Extended Data Fig. S6** and **S8**. **Regions 1 and 2** are in the granular layer, where tau is low in fibrillar content as indicated by μXRD. **Regions 3 and 4** are in the hilus where lesions are dominated by fibrillar tau. The white arrows in **Regions 3 and 4** highlight the fibrillar tau identified by the 4.7 Å, β-strand reflection. The relative intensity of colour scale bars is given in arbitrary units. The relative intensity is proportion to the mass fraction of elemental deposition.

Simultaneous collection of μXRF and μXRD data allows correlation of elemental distributions with tau accumulation and fibrillation. The 2.5 μm μXRF images for calcium (Ca), zinc (Zn), iron (Fe) and copper (Cu) have been mapped to associate the ROI scanned by μXRD (Fig. 4). Both zinc and calcium (Fig. S6) accumulate in essentially all tau lesions whether tau fibrils are present or not. To get better detection of low-Z elements, such as sulfur (S) and phosphorous (P), additional μXRF maps were acquired at the ID21 beamline, which operates under vacuum to make possible the use of low-energy X-rays (2.1–11 keV) required to detect these elements (see details in the methods section) (Fig. 5 and Fig. S8). Fig. 5 is a comparison of the distribution of Zn, P, Ca, and S (as determined by μXRF) with the presence of tau aggregates and fibrils in four regions as designated in Fig. 2d. In this Figure, **regions 1 and 2** are in the granular layer, where tau is low in fibrillar content and **regions 3 and 4** are in the hilus where lesions are dominated by high fibrillar tau. Like zinc and calcium, phosphorous appears to co-locate with tau independent of fibrillization state. In contrast, the deposition of sulfur (S) appears to be greater in lesions containing fibrillar tau than in lesions in the granular layer that have relatively lower fibrillar content. This suggests a correlation of tau fibrillation with S accumulation.

## 4. Discussion

### 4.1. The level of tau fibrillization associated with neuronal cellular type

A combination of immunohistochemistry and scanning μXRD has been used to assess the distribution of tau aggregate types across a single section of human brain tissue, revealing that the level of tau fibrillization within a lesion correlates with its gross morphological characteristics. While electron microscopy (and cryo-electron microscopy of isolated tau filaments) have demonstrated the presence of fibrillar tau in Pick bodies (Wiśniewski et al., 1972), our observations indicate that the content of fibrillar tau in Pick bodies in the granular layer is relatively low and that most of the tau present must be in disordered or amorphous aggregates or fibrillar fragments too small to be detected by X-ray scattering. By contrast, fibrillar structures within typical AD related neurofibrillary tangles present in pyramidal cells are readily detected by this same technique. This correlation between fibrillar tau and neuronal cellular type was also identified in the analysis of diffuse and sharp 4.7 Å component in wide–angle scattering regime (Fig. S9). A clearer visualization of the fibrillar distribution is shown in Fig. S9b. These observations have been replicated in another case with Pick’s disease, shown in Figs. S10–S13. Thus, our μXRD studies indicate that the density of fibrils within Pick bodies is far lower than in NFTs which is in accord with the observation that Pick bodies are less prominently stained with β-pleated sheet-specific dyes like Thioflavin S or Congo Red (Falcon et al., 2018). Within NFTs, fibrils are packed so tightly that they form domains with a relatively well-ordered hierarchical organization as detected in our SAXS data, This dense packing may influence the accumulation or exclusion of other macromolecular species or elements, thereby affecting the microenvironment within the lesions.

### 4.2. Ionic accumulation influences tau aggregation

Recent research has suggested that ionic imbalance may promote protein aggregation and oxidative stress in the central nervous system in neurodegenerative diseases. (Kim et al., 2018) For example, biometal ions have been considered to have effects on Aβ and tau aggregation. Calcium and zinc, have been found to co-locate with Aβ within plaques of the brains from AD subjects. (Miller et al., 2006) Iron and copper have also been found to co-locate with Aβ and tau aggregations in AD. (Kim et al., 2018; Li et al., 2017) Abnormal biometal homeostasis also causes protein aggregates in Parkinson’s disease. While Lewy Bodies have been found to have a higher concentration of iron and copper than surrounding tissue, aggregates of superoxide dismutase 1 (SOD1) have enriched iron content but reduced copper. (Genoud et al., 2020) The association between deficient copper and SOD1 mutations has also been observed in amyotrophic lateral sclerosis (ALS). (Bourassa et al., 2014) However, the correlation between metal ion deposition and tau aggregates in Pick’s disease has been rarely studied.

Our μXRF microscopy observations show that some elements accumulate in tau-containing lesions at higher concentrations than in the surrounding tissue in Pick’s Disease. Tissue fixation and preparation may wash out elements not bound to immobilized species, suggesting that most detected elements are specifically bound to macromolecular constituents. Levels of zinc, calcium, iron and phosphorous appeared higher in lesions than in the surrounding tissue, with no overt preference for lesion type. In contrast, no accumulation of sodium, magnesium and aluminum was observed in any tau-containing lesion. Interestingly, copper, which is thought to induce Aβ and tau aggregates in AD, shows no obvious accumulation in lesions containing either fibrillar or nonfibrillar tau. Accumulation of phosphorus in tau-containing lesions was expected due to the role that hyper-phosphorylation of tau appears to have in disease progression. μXRF imaging confirms the accumulation of phosphorus in essentially all tau lesions examined with no apparent preference for lesions with higher or lower levels of tau fibrillation. This suggests that phosphorylation of tau may play a passive role in tau fibril assembly. In contrast, sulfur appears to accumulate to higher levels in fibrillar lesions than in lesions with low levels of fibrillar tau, consistent with the hypothesis that sulfur-containing compounds may play a role in tau fibrilization (Goedert et al., 1996). Sulfated glycosaminoglycans are known to induce the formation of tau filaments *in vitro*. (Goedert et al., 1996; Hasegawa et al., 1997) Observation of a correlation of tau fibrillation with high sulfur deposition *in situ* provides support for the hypothesis that, consistent with *in vitro* observations, glycosaminoglycans may actively promote tau fibrillation in tissue. (Hasegawa et al., 1997; Nishitsuji and Uchimura, 2017; Lewkowicz et al., 2021)

The current data highlight differential association of several elements – both positively and negatively charged - with either Pick bodies or neurofibrillary lesions, with implications for their contribution to tau aggregation in different cellular milieus. The ability to map specific aggregation states of tau, as demonstrated here, offers a new approach to studying tau distribution and structural polymorphism.

## 5. Conclusions

The correlation between tau fibrillation levels, elemental deposition, and gross lesion morphology suggests that the biochemical milieu within tau lesions is influenced not only by the accumulation of tau but also by the degree of fibrillar tau formation. Similar to how biological condensates create local microenvironments that promote distinct biochemical processes (Banani et al., 2017), high concentrations of tau may alter the overall composition of lesions. This is particularly evident for elements traceable by μXRF, as demonstrated in our study. While it is unsurprising that the constituents of a lesion differ from the surrounding tissue, the different elemental composition of lesions containing fibrillar *versus* non-fibrillar tau aggregates might be considered unexpected. These observations demonstrate a correlation of lesion morphology with anatomical localization, degree of tau fibrillation and differential accumulation of elements and suggest that lesions containing different levels of tau fibrils harbor biochemically distinct microenvironments that influence both lesion morphology and tau seed formation and spreading.

## Supplementary Material

1

Supplementary data to this article can be found online at https://doi.org/10.1016/j.nbd.2025.107104.

## Data Availability

Datasets supporting the current study will be shared by the lead contact upon request. Any additional information required to reanalyze the data reported in this paper is available from the lead contact upon request.

## Data availability

Further information and requests for resources and reagents should be directed to and will be fulfilled by the lead contact, Jiliang Liu, (jiliang.liu@esrf.fr).

## Abbreviations:

AD: Alzheimer’s disease
CA4: Cornu Ammonis region 4
CA3: Cornu Ammonis region 3
cross-beta: prototypical conformation of neuropathological fibrils in which protein beta strands extend perpendicular to the axis of the fibril
cryoEM: cryogenic electron microscopy
DG: dentate gyrus
ESRF: European Synchrotron Radiation Facility
FTD: frontotemporal dementia
FTLD: tau frontotemporal lobar degeneration with tau pathology
FWHM: full-width/half maximum
ID13: x-ray beam line 13 at the European Synchrotron Radiation Facility
ID21: x-ray beam line 21 at the European Synchrotron Radiation Facility
MADRC: Massachusetts Alzheimer’s Disease Research Center
μXRD: micro-x-ray diffraction
μXRF: micro-X-ray fluorescence
NFT: neurofibrillary tangle
NPF: narrow Pick filament
Q: momentum transfer related to x-ray scattering angle by Q = 4π sin(θ)/λ where 2θ is the scattering angle and λ is the X-ray wavelength. Roughly proportional to x-ray scattering angle
ROI: region of interest
WPF: wide Pick filament
XRF: x-ray fluorescence
XRD: x-ray diffraction
