# Supplementary material for: Molecular polymorphism of tau aggregates in Pick’s disease
Source: Neurobiol Dis. Author manuscript; Available in PMC 2025 Nov 25. (PMC12645749; doi:10.1016/j.nbd.2025.107104)
Supplement: 1 [file NIHMS2120878-supplement-1.docx]

**Supplementary Material: Molecular Polymorphism of tau aggregates in Pick’s disease**

Jiliang Liu^a*^, Theresa Connors Stewart^b^, Derek H. Oakley^b,c,d,e^, Bradley T. Hyman^b,e^, Manfred Burghammer^a^, Marine Cotte^a^, Lee Makowski^f^*.

a. European Synchrotron Radiation Facility, Grenoble 38043, France;

b. Massachusetts Alzheimer’s Disease Research Center, Boston, MA, USA;

c. Department of Pathology, Massachusetts General Hospital, Boston, MA,USA;

d. C.S. Kubik Laboratory for Neuropathology, Massachusetts General Hospital, Boston, MA, USA;

e. Department of Neurology, Harvard Medical School, Boston, MA, USA;

f. Bioengineering Department, Northeastern University, Boston, MA 02115, USA.

## **Fitting SAXS profile from high fibrillar tau**

The SAXS regime contains information on structural features of the scattering particles that have characteristic dimensions in the 1-100 nm scale. At this level, SAXS can be considered as scattering from a continuous distribution of electron density and approximated by a simplified model. Based on the structure of cross-β tau filament of Pick’s disease as determined by cryoEM.[1,2] we constructed a parallelepiped model as shown in **Figure S3a**, with a cross section of 10 x 100 Å for the ‘narrow Pick filament’ (NPF) and 10 x 200 Å for the ‘wide Pick filament’ (WPF). The form factor of a parallelepiped can be calculated as:

$$F\left( q \right)=\frac{sin\left( q\frac{A}{2} \right)}{q\frac{A}{2}}\frac{sin\left( q\frac{B}{2} \right)}{q\frac{B}{2}}\frac{sin\left( q\frac{C}{2} \right)}{q\frac{C}{2}}$$

Considering the centroid symmetry of the parallelepiped, the 1D intensity profile can be calculated as the spherically averaged squared form factor as:

$$I\left( q \right)=\left\langle{F\left( q \right)}^{2} \right\rangle$$

$$=scale*\int_{0}^{\pi} \int_{0}^{2\pi} \left[ \frac{sin\left( q\frac{A}{2}sin\phi sin\varphi\right)}{q\frac{A}{2}sin\phi sin\varphi}\frac{sin\left( q\frac{B}{2}cos\phi sin\varphi\right)}{q\frac{B}{2}cos\phi sin\varphi}\frac{sin\left( q\frac{C}{2}cos\varphi\right)}{q\frac{C}{2}cos\varphi} \right]^{2}sin\left( \varphi\right)d\phi d\varphi$$

………………………..1

The slope of the SAXS intensity provides an approximation for the dimensions of these filaments. **Figure S4 a)** and **b)** show a parallelepiped model with transverse dimension of 10 x 100 Å (A, B direction in **Figure S4a**) and 150 Å in longitudinal direction (C direction in **Figure S3a**) that gives rise to a profile with a slope comparable to the SAXS pattern from fibrillar tau (**Figure S3c**). This structural model is consistent with the NPF model of tau filament for Pick’s disease.[1] However, the features of the SAXS intensity pattern at 0.07 Å^-1^ and 0.2 Å^-1^ (**Figure 3**) indicate that, *in situ*, tau filaments further aggregate to form larger scale hierarchical structures within the lesion. The interference scattering arising from this higher order structure can be calculated as:

$$I\left( q \right)= \left\langle\left[ F(q)(\sum_{i=0}^{N} \sum_{j=0}^{M} S_{ij}\left( q \right)) \right]^{2} \right\rangle$$

where $S_{ij}\left( q \right)=\int\delta\left( \vec{x}_{ij} \right)e^{\vec{q}\vec{x}}d\vec{x}=e^{\vec{q}\vec{x}_{ij}}$ is the Fourier transform of the translation vector in real space. Thus, the spherically averaged intensity profile for the model of higher order aggregation of tau, shown in the Figure **S3c**, is calculated as:

$$I\left( q \right)=scale*\int_{0}^{\pi} \int_{0}^{2\pi} \left[ \frac{sin\left( q\frac{A}{2}sin\phi sin\varphi\right)}{q\frac{A}{2}sin\phi sin\varphi}\frac{sin\left( q\frac{B}{2}cos\phi sin\varphi\right)}{q\frac{B}{2}cos\phi sin\varphi}\frac{sin\left( q\frac{C}{2}cos\varphi\right)}{q\frac{C}{2}cos\varphi}\left( 1+e^{\vec{q}\vec{x}_{1}}+e^{\vec{q}\vec{x}_{2}}+e^{i\vec{q}\vec{x}_{3}} \right) \right]^{2}sin\left( \varphi\right)d\phi d\varphi$$

………………………..2

where x1= [30, 0, 0], x2 = [80, 0, 0,] and x3= [110, 0, 0]. **Figure S3d** shows that a SAXS profile calculated from a model including four parallelepiped fibrils with 10 x100 Å transverse structure and 20Å in the axial direction exhibits interference scattering consistent with experimental SAXS in both maximum position and log-log slope. However, the predicted sharpness of the calculated intensity is far greater than experimental data, suggesting polymorphism in the higher-order structural organization. The polymorphism in the the higher-order organization of tau fibrils was incorporated into the model as:

$$I\left( q \right)=\iint{p\left( d1,d2 \right)I}_{d1d2}dp$$

$$p=\frac{1}{N_{d_{1}}N_{d_{2}}}$$

where d1 was given values of [ 20Å, 25Å, 30Å, 35Å, 40Å] and d2 [ 85Å, 92.5Å, 100Å, 107.5Å, 115Å]. The result of this integration is shown in **Figure S4**, which demonstrates that this results in a model for the hierarchical organization of tau in these NFTs that is consistent with observation.

SAXS scattering includes contributions from other constituents of the lesions as well as that due to voids formed during dehydration of the tissue.[3] In many cases, the contribution of other constituents of lesions to the observed scattering is closely similar to that observed in scattering from adjacent tissue. This ‘background’ scattering from tissue provides an estimate of the contribution of other constituents to that observed from lesions.

## **Fitting μXRF**

At high X-ray energy (13keV), the XRF spectrum of heavier elements was collected simultaneously with scanning μXRD. **Figure 1c** exhibits the averaged spectrum from three regions, one identified as tissue, one as low fibrillar tau and one as high fibrillar tau by the integral intensity of β-strands scattering at 1.36 Å^-1^ as detailed in **Figure 1b**. Qualitatively the density of sulfur (S), calcium (Ca), iron (Fe) and zinc (Zn) in the irradiated volume can be obtained by fitting the spectrum with elements determined from **Figure S5**.

**Correlating μXRF and μXRD**

**Figure S6** includes a superposition of the map of scattering of β-strands (from μXRD) with the maps of Zn and Ca. The map of scattering of β-strands has been normalized and transformed to an RGB image with μXRD as red channel and Zn as green channel, Ca as blue channel. In **Figure S6b** regions corresponding to the deposition of fibrillar tau has a white color, which indicates that the presence of high fibrillar tau with β-strands is associated with deposition of both Zn and Ca. In the Granular layer the strong correspondence of Zn and Ca with low fibrillar tau appears as cyan (light blue) instead of white.

## **The analysis of high resolution XRF**

High resolution XRF image data was obtained by scanning the tissues with a 0.3 × 0.8 μm beam at ID21 ESRF using X-rays with an energy of 4 KeV to emphasize the signal from light elements such as phosphorous, sulfur and calcium. Data was collected on a square grid of 99 x 99 μm^2^ with step size of 1 μm^2^. A complete XRF spectrum such as that shown in **Figure S7** is collected at each scan position. The relative abundances of elements at each position are determined by fitting the XRF spectra with multi-Gaussian functions using PyMca software.

Due to differences in experimental setup, the registration of the high-resolution maps of calcium, phosphorous and sulfur in regions R1 to R4 vary slightly from that shown in **Figure S8**. However, the morphologies of cells, particularly those containing fibrillar tau, enabled the alignment of the high-resolution XRF maps with those in R1 to R4 in **Figure S8**. The high resolution XRF maps exhibit more details of the distribution of elements within the cell. Superposition of maps in **Figure S8** demonstrates that calcium and zinc content co-locate with both low-fibrillar and high-fibrillar tau. Region R4 of **Figure 5** and **Figure S8** shows that sulfur co-locates with fibrillar tau. Overlap of the maps of calcium, phosphorous and sulfur demonstrates that calcium and phosphorous are distributed throughout the pyramidal cells, which appears bright white in Region 3 and 4 in **Figure S8**.

Supplementary Figures


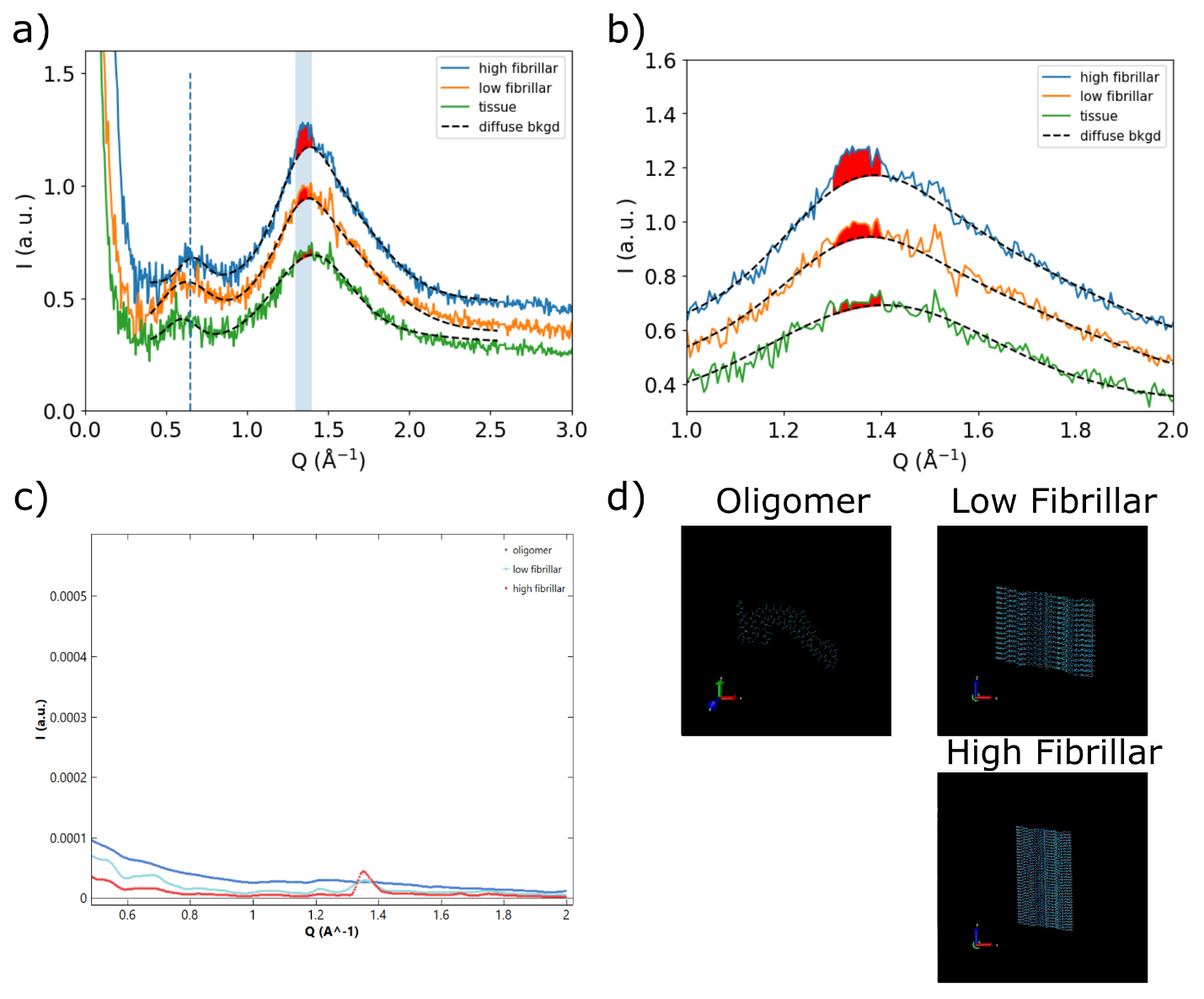


**Figure S1.** **a)** Comparison of diffraction from regions of a histological section containing only tissue (green), containing lesions dominated by low-fibrillar tau (orange) and containing lesions composed of fibrillar tau (blue). The smooth background curves (dashed lines) are calculated as a sum of Pseudo-Voigt functions and represent an estimate of the scattering of tissue which is comprised largely of fixed, partially denatured macromolecules. The vertical dashed line at Q = 0.65 Å^-1^ indicates the peak arising from structural features with characteristic dimension of ~ 10 Å. The blue vertical bar highlights the Q range from 1.32 Å^-1^ to 1.4 Å^-1^, which corresponds to structural features with dimensions ~ 4.7 Å. **b)** the deposition of tau enhances the intensity at ~ 1.32 Å^-1^, with progressively greater levels of fibrillation giving rise to a sharper and more intense peak at that position. Scattering from low-fibrillar tau causes the broad, wide-angle peaks to shift from Q = 0.5 Å^-1^ to 0.65 Å^-1^ and Q = 1.42 Å^-1^ to 1.36 Å^-1^. The degree of fibrillation is estimated by subtraction of the diffuse background, resulting in a difference intensity (red) indicative of the degree of tau fibrillation. The broad scattering peak in the wide-angle regime spanning a region from 1.0 < Q < 2.0 Å^-1^ is generated by scattering from disordered, partially denatured and cross-linked macromolecules that make up much of the mass of the fixed tissue.


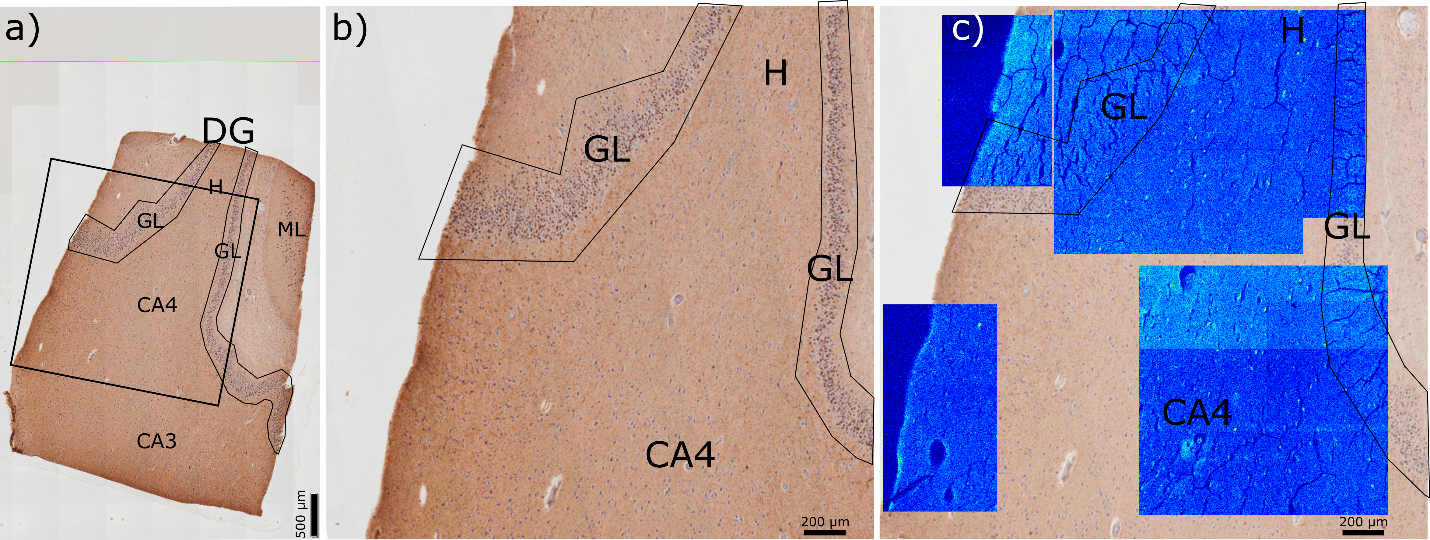


**Figure S2.** **a)** An immunostained serial section of tissue from Dentate Gyrus providing a larger anatomical context for the images in **Figure 2** and exhibiting the distribution of tau and its association with tissue morphology. **b)** Enlargement of regions scanned by X-ray microdiffraction. **c)** Maps of the distribution of fibrillar tau determined by the intensity of the 4.7 Å peak (estimated as described in **Figure S1**) superimposed on the image of the immunostained serial section. Lighter blue indicates greater integral intensity. Additional analyses of these ROIs are detailed in **Figure 2**. Abbreviations in the figure: **GL**-Granular Layer, **H**-Hilus, **CA4**- Cornu Ammonis region 4.


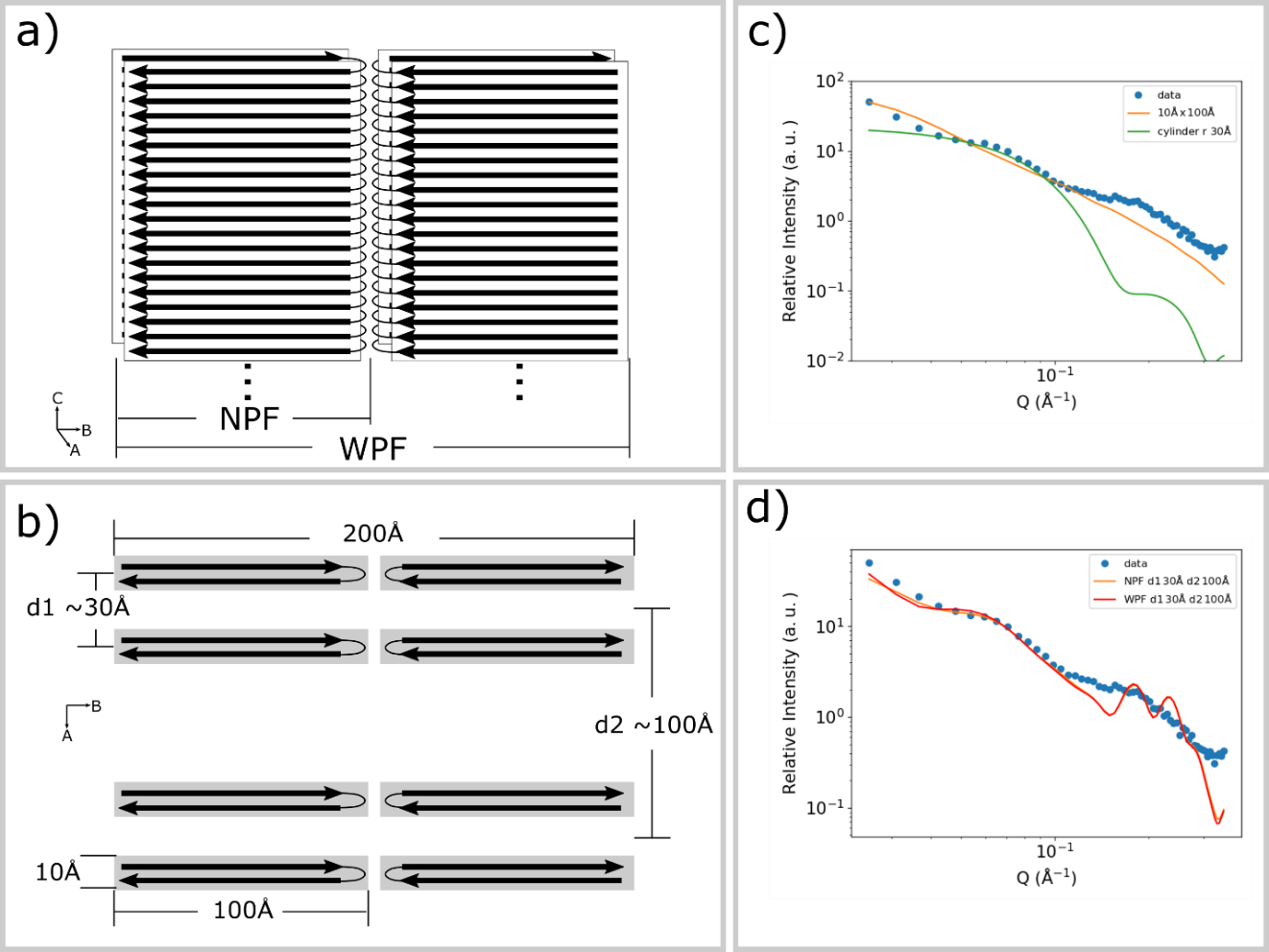


**Figure S3.** Constructing a hierarchical model of fibrillar Tau to fit the observed SAXS data. a) The fibril model is built on the basis of the structure of narrow fibrils (NF) determined by cryoEM. The wide fibrils are comprised of two narrow fibrils related to one another by a two-fold axis parallel to the fiber axis. b) Models constructed as hierarchical aggregates of narrow fibrils, including wide fibrils, exhibit organization on at least two length scales. The first is formed from the intra-fibril distance, d1, of ~ 30 Å and the second from inter-fibril distances, d2, of ~ 100 Å. c) SAXS calculated from the Narrow Fibril model (red) exhibits a slope comparable to that observed, but lacks the distinctive shoulders. A cylinder model (green curve) proposed earlier [4], reproduces some of the SAXS features but does not predict the overall decrease of intensity as a function of Q. d) The calculated SAXS profile for these choices of d1 and d2 fits the first intensity shoulder well, but not the second.


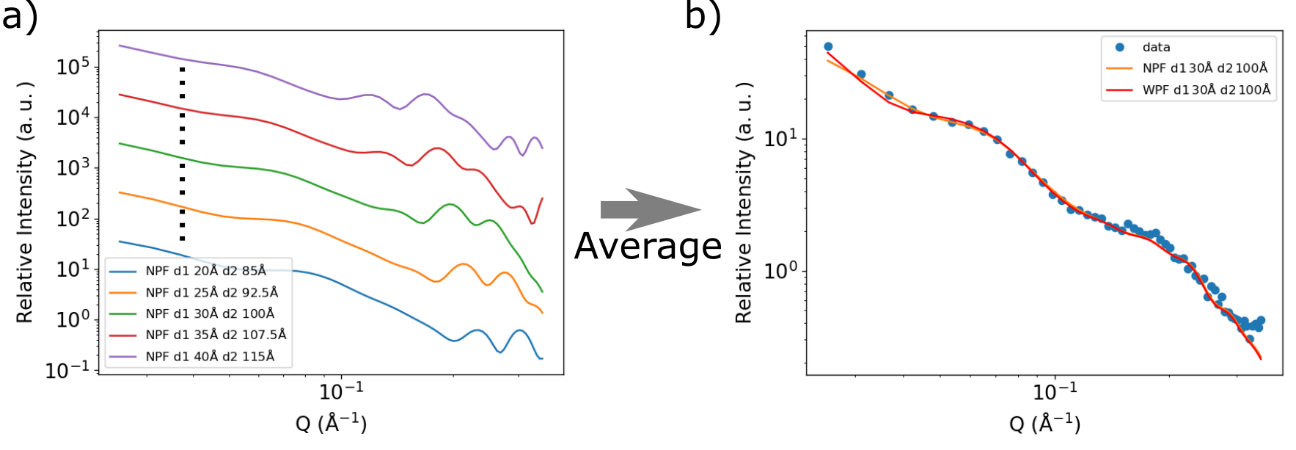


**Figure S4.** Adding polydispersity to the multi-scale hierarchical model for Tau filaments. a) For any given choice of d1 and d2, the calculated SAXS profiles include features not consistent with experiment. b) An ensemble of fibrillar aggregates constructed with d1 varying from 20 – 40 Å and d2 from 85 to 115 Å with equal probability, results in a SAXS pattern consistent with that observed *in situ* strongly suggesting that *in situ* the fibrillar aggregates are organized in a hierarchical structure that exhibits limited variation of fibril-fibril distances.


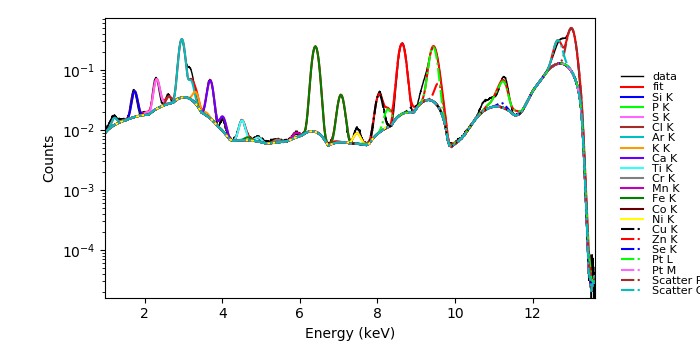


**Figure S5**. The averaged XRF spectrum from ID13 µXRF image map, with peak heights of individual elemental peaks estimated using PyMca


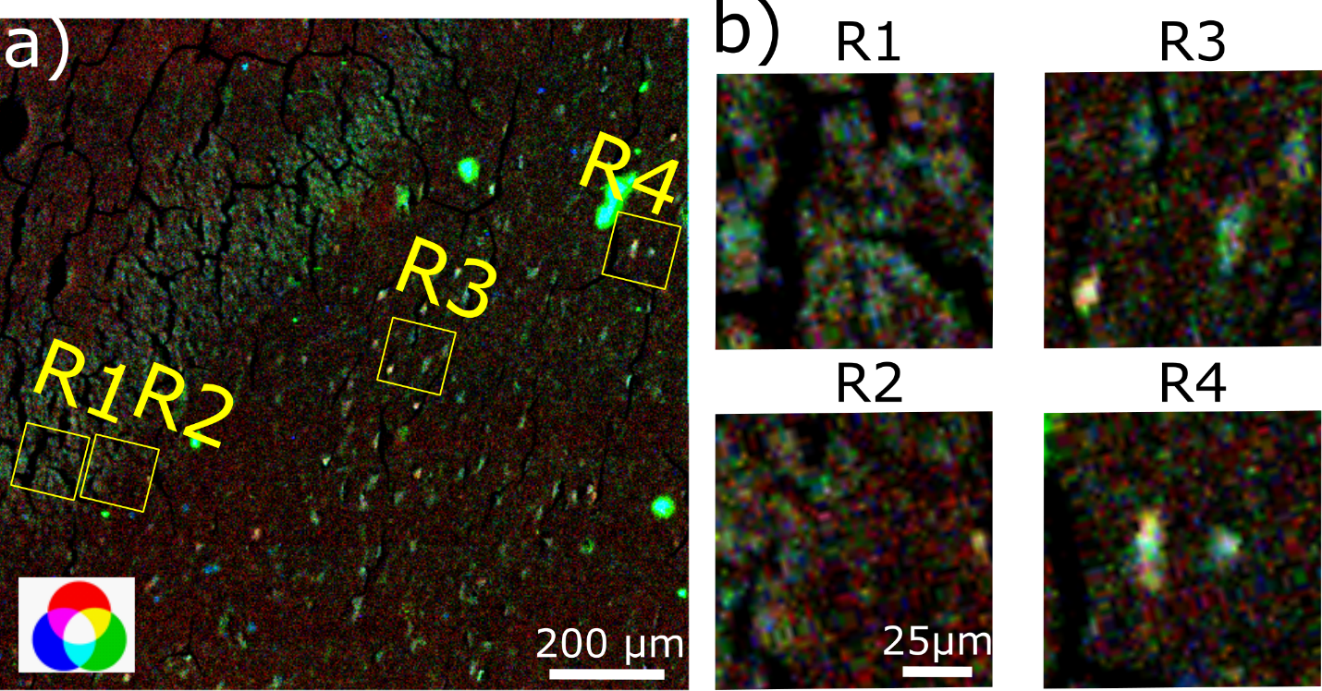


**Figure S6**. Superposition of the distribution of total tau estimated from the scattering at 4.7 Å with the distribution of Zn and Ca from XRF. (a) includes the entire ROI exhibited in **Figure 2**. (b) includes enlargements of four regions within that ROI. R1 and R2 in the granular layer exhibit strong co-deposition of Zn and Ca with low-fibrillar tau as visualized by the superposition of green and blue leading to cyan or light blue color in the enlargements on the right. In R3 and R4, in the hilus, places where fibrillar tau co-localizes with Zn and Ca appear as white. The label in **a)** shows the mixture of RGB colors.


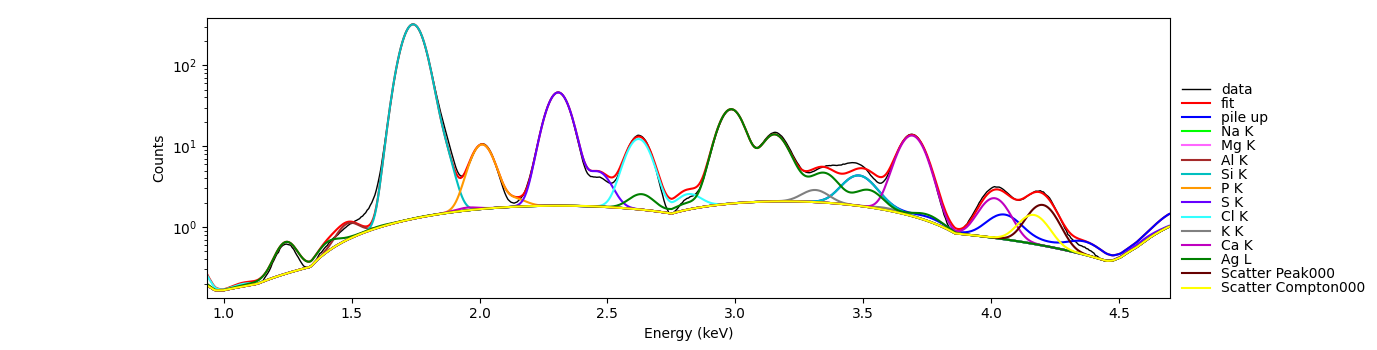


**Figure S7.** The XRF spectrum from ID21 µXRF image map, fitted using PyMca


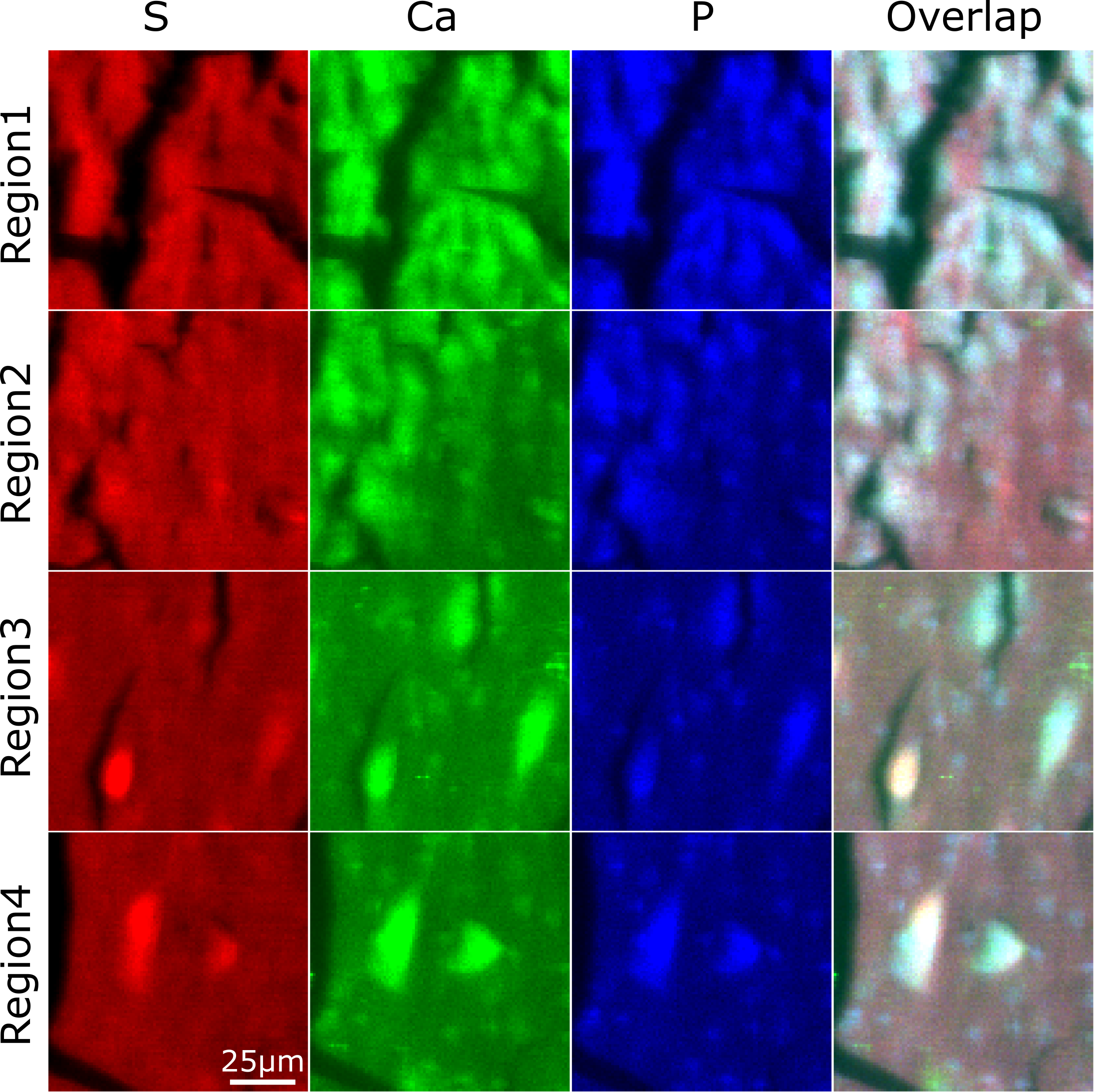


**Figure S8**. Comparison of the distribution of elements from high-resolution XRF data. Here, the maps in **Figure 5** of main text are transformed to RGB format. The map of sulfur is transformed to the red channel, calcium to the green channel, and phosphorous to the blue channel. Both calcium and phosphorous exhibit deposition in all tau-containing lesions. Sulfur demonstrates strong correlation with calcium and phosphorous only in Regions 3 and 4, indicating a strong co-localization with fibrillar tau.


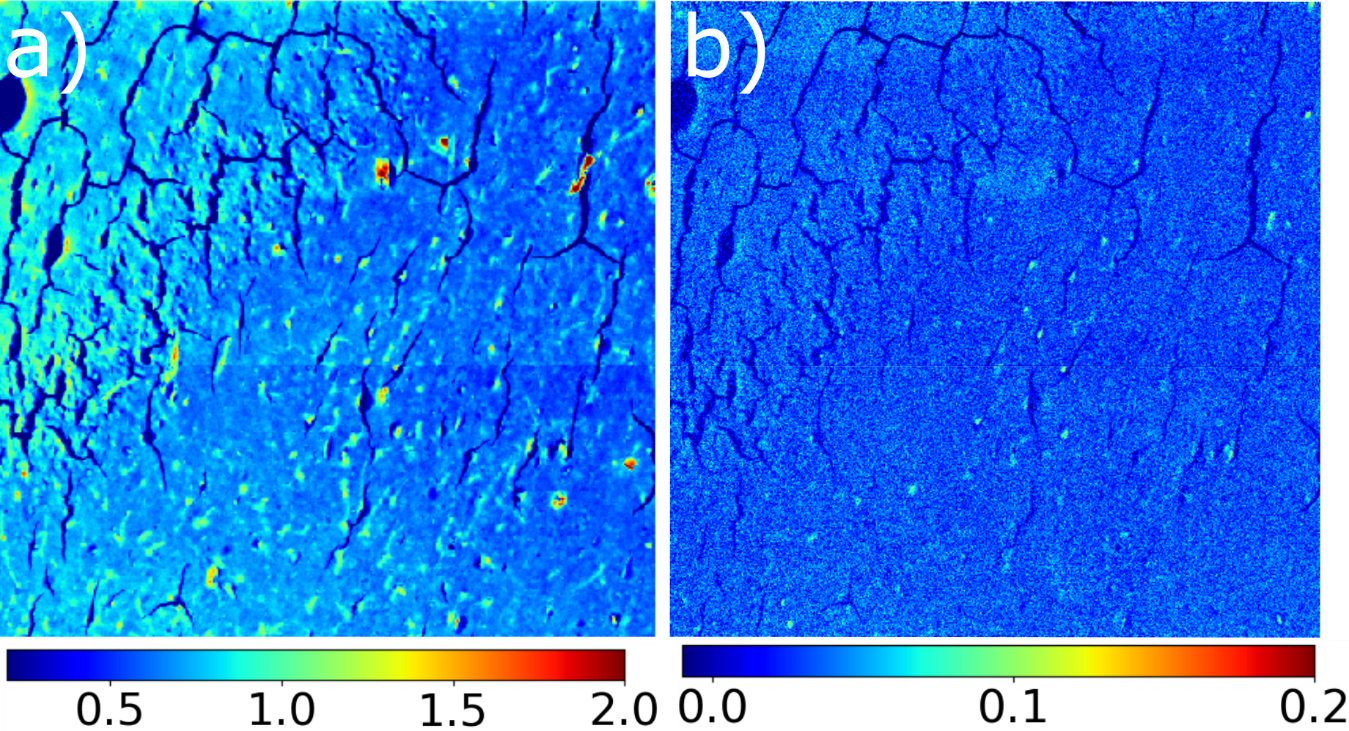


**Figure S9. a)** The map of the integral intensity of scattering from the Q range from 1.32 Å^-1^ to 1.4 Å^-1^ allows visualization of the distribution of molecular mass for the proteinaceous tissue in the Dentate Gyrus. The distribution includes many peaks that correspond to tau-containing lesions but does not distinguish between lesions with low or high levels of fibrillar tau. **b)** the distribution of fibrillar tau in the tissue as estimated by subtraction of diffuse tissue scattering as detailed in **Figure S1**. Lighter blue indicates greater concentration of fibrillar tau. Fibrillar tau is concentrated in punctate features prominent in (b) and is largely absent from the granular layer that occupies the upper right in these images.


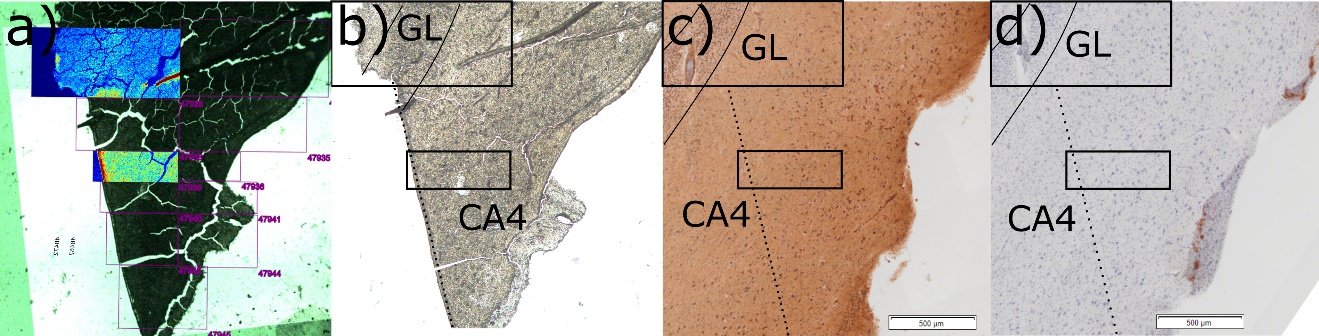


**Figure S10.** Images of a tissue sample from Pick’s disease subject 2. (a) Microscope image after uXRD measurement with regions measured by uXRD indicated by rectangles. (b) Microscope image before uXRD measure, used for registration of the images of serial sections immunostained for tau and Aβ. (c) Microscope image of a serial section immunostained for tau. (d) Microscope image of a serial section immunostained for Aβ. Registration of the images of x-ray irradiated tissue and immunostained serial sections is challenging due to distortion of tissue during staining. Regions where registration is of high confidence are highlighted by the dashed lines in the images. These images demonstrate that the regions of uXRD scanning extend across both granular layer and CA4.


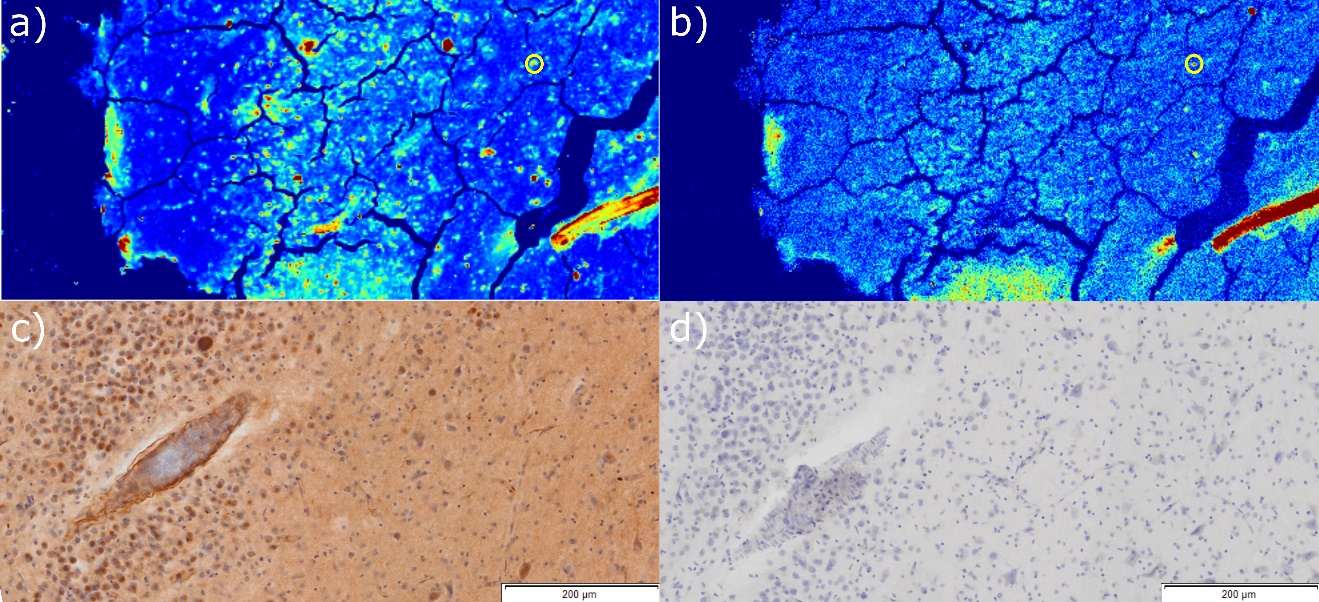


**Figure S11.** Maps of low- and high-fibrillar tau in subject 2 corresponding to the inset in Figure S10:: The scanned region covered both GL and CA4. (a) The SAXS integral intensity map, indicating the mass density within the tissue demonstrates the high density of protein deposition within Pick bodies. The granular layer can be identified as a strip exhibiting high intensity SAXS. (b) The intensity map of 4.7 Å reflections after background subtraction, demonstrating the locations of fibrillar protein deposits which are largely confined to CA4 and absent from the granular layer. The yellow circles highlighted the plaques containing high fibrillar tau. (c) Microscope image of a serial section immunostained for tau. Tau is abundant in the granular layer and exhibits significant deposition in Pick bodies. (d) Microscope image of a serial section immunostained for Aβ. No obvious deposition of Aβ was observed.


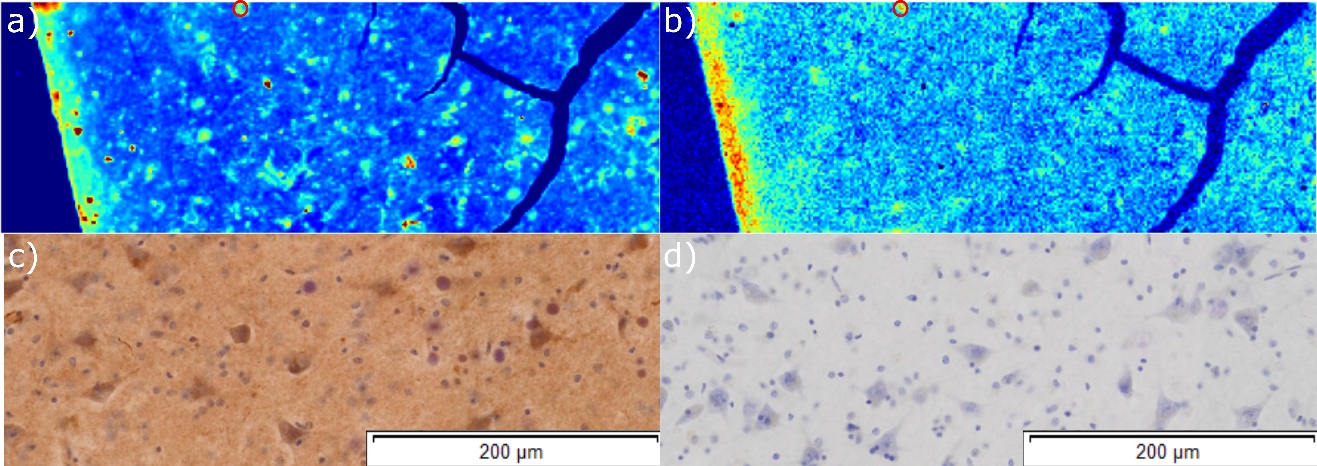


**Figure S12.** Scans of CA4 from subject 2: (a) SAXS integral intensity map, indicating the mass density within the tissue. Neurons with round and elongated morphology are observed. (b) The intensity map of 4.7 Å reflections after background subtraction. The red circles highlight the plaques containing low fibrillar tau. (c) Microscope image of a serial section immunostained for tau. Neurons containing Pick Bodies and neurofibrillary tangles were observed. (d) Microscope image of a serial section immunostained for Aβ. No obvious Aβ deposits were observed.


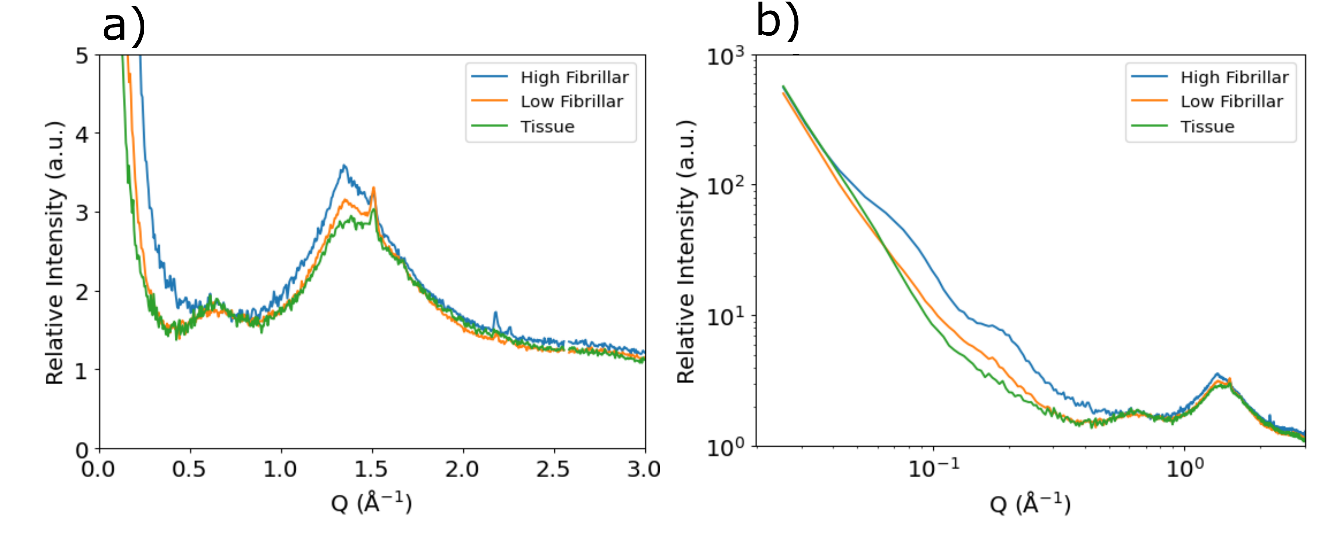


**Figure S13.** Scattering from tissue, non-fibrillar and fibrillar deposits from subject 2 in the WAXS regime (a) and SAXS regime (b): The intensity profile from high fibrillar tau (blue curve) shows a significant sharp peak at 4.7 Å (q = 1.34 Å^-1^), which is accompanied by strong, distinct SAXS features that appear as undulating shoulders on the strong SAXS scattering. This pattern was derived from the NFT within the yellow circle in Figure S11. Scattering from low fibrillar tau (orange line) shows a moderate peak at 4.7 Å but only the slightest hint of shoulders in the SAXS regime. The intensity profile for tissue background (green) contains none of these features in either the WAXS or SAXS regimes.

Reference:

1. Falcon B, Zhang W, Murzin AG, Murshudov G, Garringer H, Vidal R, et al. Structures of filaments from Pick’s disease reveal a novel tau protein fold. Nature. 2018;561(7721):137-40.

2. Fitzpatrick AW, Falcon B, He S, Murzin AG, Murshudov G, Garringer HJ, et al. Cryo-EM structures of tau filaments from Alzheimer’s disease. Nature. 2017;547(7662):185-90.

3. Nepal P, Bashit AA, Makowski L. Characterization of sub-micrometre-sized voids in fixed human brain tissue using scanning X-ray microdiffraction. J Appl Crystallogr. 2024. DOI: 10.1107/S1600576724008987.

4. Schweers O, Schonbrunn-Hanebeck E, Marx A, Mandelkow E. Structural studies of tau protein and Alzheimer paired helical filaments show no evidence for beta-structure. J Biol Chem. 1994;269(39):24290-7.
